# Comparative effects of exercise type and dose on depression in children and adolescents: a network meta-analysis

**DOI:** 10.3389/fpsyg.2025.1632111

**Published:** 2025-08-13

**Authors:** Xiaofeng Cao

**Affiliations:** Department of Sport and Leisure Studies, Namseoul University, Cheonan, Republic of Korea

**Keywords:** exercise, depressive symptoms, adolescents, network meta analysis, resistant training

## Abstract

**Background:**

This study seeks to evaluate the comparative effectiveness of various physical activity modalities: including aerobic (AT), resistance (RT), flexibility (FT), and combined aerobic-resistance (AT + RT) training, in reducing depressive symptoms among children and adolescents, with the aim of determining the most effective type and dosage for optimizing mental health outcomes in this population.

**Methods:**

Following a comprehensive search of PubMed, MEDLINE, Embase, PsycINFO, the Cochrane Central Register of Controlled Trials (CENTRAL), Web of Science, and other databases, studies were selected according to stringent inclusion and exclusion criteria. Quality assessment, data extraction, and subsequent analysis were conducted using RevMan 5.3 and Stata 16.0 software.

**Results:**

Seventeen high-quality studies, involving 1,357 young participants, were included in this meta-analysis to explore the impacts of the four exercise types on depressive symptoms. Network Meta-Analysis results indicated that RT (SMD = −0.52, 95% CI: −0.95 to −0.09) were significantly more effective than AT (SMD = −0.40, 95% CI: −0.56 to −0.25) and AT + RT (SMD = −0.30, 95% CI: −0.49 to −0.10) in reducing depressive symptoms (*p* < 0.05). We found that exercising for 20 ~ 30 min per session (SMD = −0.35, 95% CI: −0.59 to −0.11), three times a week (SMD = −0.42, 95% CI: −0.67 to −0.16), over a 6 ~ 8 week period (SMD = −0.74, 95% CI: −0.95 to −0.52) yielded the most significant reductions in depressive symptoms (*p* < 0.01).

**Conclusion:**

Different exercise types, including AT, RT, and AT + RT, can effectively reduce depressive symptoms in children and adolescents, with RT emerging as the most effective approach. It is recommended that children and adolescents participate in physical activity at least three times weekly for 6 ~ 8 weeks, with each session lasting 20 ~ 30 min. Greater frequency and duration may lead to even more substantial improvements in depressive symptoms.

## 1 Introduction

Adolescent mental health issues have become a major global public health concern. According to the World Health Organization (2021), one in seven people between the ages of 10 and 19 suffers from a mental disorder, and depression, anxiety, and behavioral disorders are the leading causes of illness and disability in this age group, accounting for 13% of the global burden of disease in this population (Orben et al., 2024). Of particular concern is the fact that untreated mental health problems can have serious consequences, and suicidal behavior triggered by mental health problems has become the fourth leading cause of death among 15- to 29-year-olds (Rocliffe et al., 2024). Adolescence is not only a period of high prevalence of mental health problems, but also a critical time for developing lifelong social and emotional skills (McGorry et al., 2024). Therefore, how to implement effective interventions during this important developmental stage has become a global concern.

Alarmingly, physical activity levels among adolescents have declined drastically worldwide. Over 80% of adolescents fail to meet the WHO’s recommendation of 60 min of daily moderate-to-vigorous physical activity (World Health Organization, 2019), with sedentary behaviors replacing active pursuits. Key physical fitness components critical for health promotion, including cardiorespiratory endurance, muscular strength, and coordination, are disproportionately affected by this decline. For example, cardiorespiratory fitness in adolescents has dropped over the past two decades (Tomkinson et al., 2019), while muscular strength deficits have been linked to increased mental health risks (Firth et al., 2018). Current exercise prescriptions for adolescents emphasize moderate intensity for 30 to 60 min, 3 to 5 times weekly, with a mix of aerobic and resistance training to target these at-risk fitness components (Schroeder et al., 2019).

Existing literature consistently highlights that a healthy lifestyle, including regular physical activity, emotion regulation, and strong interpersonal and problem-solving skills is a key protective factor for adolescent mental health (Bailey et al., 2018). Among these factors, physical activity has been widely recognized as a particularly effective intervention for alleviating depressive symptoms in adolescents (Ma et al., 2023). Despite growing awareness, a substantial global treatment gap remains: many mental health issues in children and adolescents are not identified or addressed early (Dastamooz et al., 2023). Research indicates that around 50% of mental health problems manifest before the age of 14, yet most affected adolescents do not receive timely or adequate treatment. Untreated depressive symptoms often persist into adulthood, negatively impacting long-term physical and mental well-being as well as life opportunities (Pollmann et al., 2023; Wasserman et al., 2023). Physical activity (PA), as a modifiable and accessible intervention, has demonstrated significant short- and long-term benefits for adolescent mental health (Psychogiou et al., 2024). Numerous studies have shown that PA is associated with reduced symptoms of depression and anxiety in adolescents (Duagi et al., 2024; Chen et al., 2024; Nusslock et al., 2024), and meta-analyses confirm its significant ameliorative effects on both anxiety and depression (Alcaraz-Ibáñez et al., 2024; Keyes and Platt, 2024; Piñeiro-Cossio et al., 2021; Li et al., 2024).

In recent years, research on exercise interventions for adolescent depression has expanded. While prior studies confirm the general efficacy of exercise (Kodal et al., 2024), critical questions remain—particularly regarding which exercise types and dosages yield the greatest benefit. For instance, some studies suggest that activities such as yoga and tai chi may be more effective than aerobic or resistance training in alleviating depressive symptoms (Luo et al., 2024). However, there is still insufficient clarity regarding optimal intensity, modality, and duration. This study aimed to systematically evaluate the effects of various exercise interventions on depressive symptoms in adolescents and to explore their dose–response relationships using a Bayesian model-based network meta-analysis. We compared the effectiveness of four common exercise types: AT, RT, FT, and AT + RT across different doses. Furthermore, we estimated the minimum effective frequency and duration required for each exercise type to improve depressive symptoms.

The findings of this study will provide robust evidence for developing more targeted and effective clinical and public health strategies to prevent and manage adolescent depression. By identifying the optimal exercise modality and dose, this research contributes to global efforts in enhancing adolescent mental health and reducing the burden of depressive symptoms.

## 2 Methods

This preregistered systematic review with network meta-analysis (PROSPERO reference number # CRD42024593375) was reported following the PRISMA checklist.

### 2.1 Study design

During the literature screening process, clear inclusion and exclusion criteria were set following the PICOS principles (i.e., patients, interventions, controls, outcomes, and study design) to ensure the scientific validity and consistency of the study.

Literature inclusion criteria: (1) Study population: There were no restrictions based on gender, race, or depression severity, as long as a validated depression scale was used. (2) Interventions: The comparison of test and control groups involved different types of exercise interventions, specifically: AT as rhythmic, low-to-moderate intensity large-muscle activity to enhance cardiovascular endurance; RT as external resistance-based activity to improve muscular strength/endurance/power; AT + RT as integrated programs with aerobic and resistance components, retaining respective intensity, duration, and frequency; and FT as low-intensity stretching and mind–body practices (e.g., yoga) to improve joint range of motion and muscle elasticity. (3) Outcome indicators: The assessment tools were all scales for measuring the severity of depression in children and adolescents, mainly the Beck’s depression inventory (BDI), Hamilton depression scale (HAMD), etc. The clinical assessment of depression severity in children and adolescents was based on these scales, with complete data acquisition for the scores.

Literature exclusion criteria: (1) Literature in non-Chinese and non-English languages; literature with inaccessible full text or data; conference abstracts, dissertations, and similar literature; (2) Studies involving animal subjects or non-child/adolescent populations; (3) Literature with non-exercise interventions or unclearly described intervention methods; (4) Literature with significant result errors due to non-standardized statistical methods or incomplete reporting of experimental data.

These inclusion and exclusion criteria ensured that the process of selecting literature was both rigorous and extensive, excluding irrelevant or incomplete studies, thus providing a high-quality evidence base for this systematic meta-analysis.

### 2.2 Search strategy

A comprehensive search was conducted in PubMed, MEDLINE, Embase, PsycINFO, CENTRAL, and Web of Science from inception to August 30, 2024, without language restrictions. Search terms included “mental disorders,” “anxiety,” “depression,” and “exercise,” “training,” or “physical activity,” among others. In addition, reference lists of relevant articles and systematic reviews were manually screened to identify potential studies. For example, We searched Web of Science using the following Boolean search strategy: (TS = ((“physical activity” OR exercise OR “aerobic training” OR “resistance training” OR “flexibility training” OR “yoga” OR “tai chi”) AND (depress* OR “depressive symptoms” OR “depressive disorder”)) AND TS = (child* OR adolescen* OR teen* OR youth OR “young people”) AND TS = (“randomized controlled trial” OR RCT OR “intervention study”)). Additionally, more detailed search strategies can be found in the Supplementary document.

All retrieved records were imported into EndNote X9 to remove duplicates. The screening process was executed in two sequential stages by two independent reviewers. First, titles and abstracts were screened against predefined inclusion and exclusion criteria to exclude obviously irrelevant studies; during this stage, each reviewer marked records as “include” “exclude” or “uncertain” based on initial information. Second, full texts of records marked “include” or “uncertain” were retrieved and assessed for final eligibility, with explicit documentation of reasons for exclusion at this stage.

Data extraction was also performed independently by the same two reviewers using a standardized extraction form, which included study characteristics, intervention details, and outcome measures. Discrepancies during screening or data extraction were resolved through discussion, and a third reviewer was consulted if consensus could not be reached. To ensure inter-rater reliability, the kappa statistic was calculated for both title/abstract screening and full-text assessment, with a threshold of *κ* ≥ 0.7 predefined to indicate substantial agreement.

### 2.3 Risk of bias evaluation of included studies

The study selection and data extraction were conducted independently by two reviewers. Titles and abstracts were first screened to exclude irrelevant studies, followed by full-text reviews to assess eligibility according to the inclusion and exclusion criteria. Any disagreements between the two reviewers were resolved through discussion or consultation with a third reviewer. Risk of bias was assessed independently and in duplicate by two investigators followed the original Cochrane RoB tool (Sterne et al., 2016). The assessment included the following domains: random sequence generation, allocation concealment, blinding of participants and personnel, blinding of outcome assessment, completeness of outcome data, selective reporting, and other potential sources of bias. According to Cochrane guidelines, studies that met all criteria were classified as low risk, those meeting some criteria as moderate risk, and those failing to meet most criteria as high risk.

### 2.4 Statistical methods

A frequentist approach was adopted for network meta-analysis, utilizing a random-effects model with inverse-variance weighting to account for between-study heterogeneity. Heterogeneity among included studies was assessed using RevMan 5.3 software, with the following thresholds: no substantial heterogeneity was indicated if *I*^2^ ≤ 50% or *p* > 0.05, while greater heterogeneity was defined as *I*^2^ > 50% or *p* < 0.05, in which case the source of heterogeneity was further explored.

Network meta-analysis was performed using a frequentist model in Stata 16.0 software, with the netmeta package and its core commands (including netmeta, netrank) for model fitting, effect size synthesis, and ranking probability analysis. Given that the outcome indicators were continuous variables and the evaluation tools varied across studies, standardized mean difference (SMD) and its 95% confidence interval (CI) were used as effect sizes to standardize results across different measurement scales.

Inconsistency thresholds: Global inconsistency across the network was evaluated using Cochran’s *Q* statistic, with a significance level set at *p* < 0.05. Local inconsistency was assessed via node-splitting analyses, comparing direct and indirect effect estimates for each intervention contrast, with *p* < 0.05 indicating significant inconsistency.

Surface Under the Cumulative Ranking Curve (SUCRA) scores were used to rank the efficacy of multiple exercise modalities, where a larger SUCRA value indicates better efficacy.

## 3 Results

### 3.1 Study characteristics

**Figure 1:**
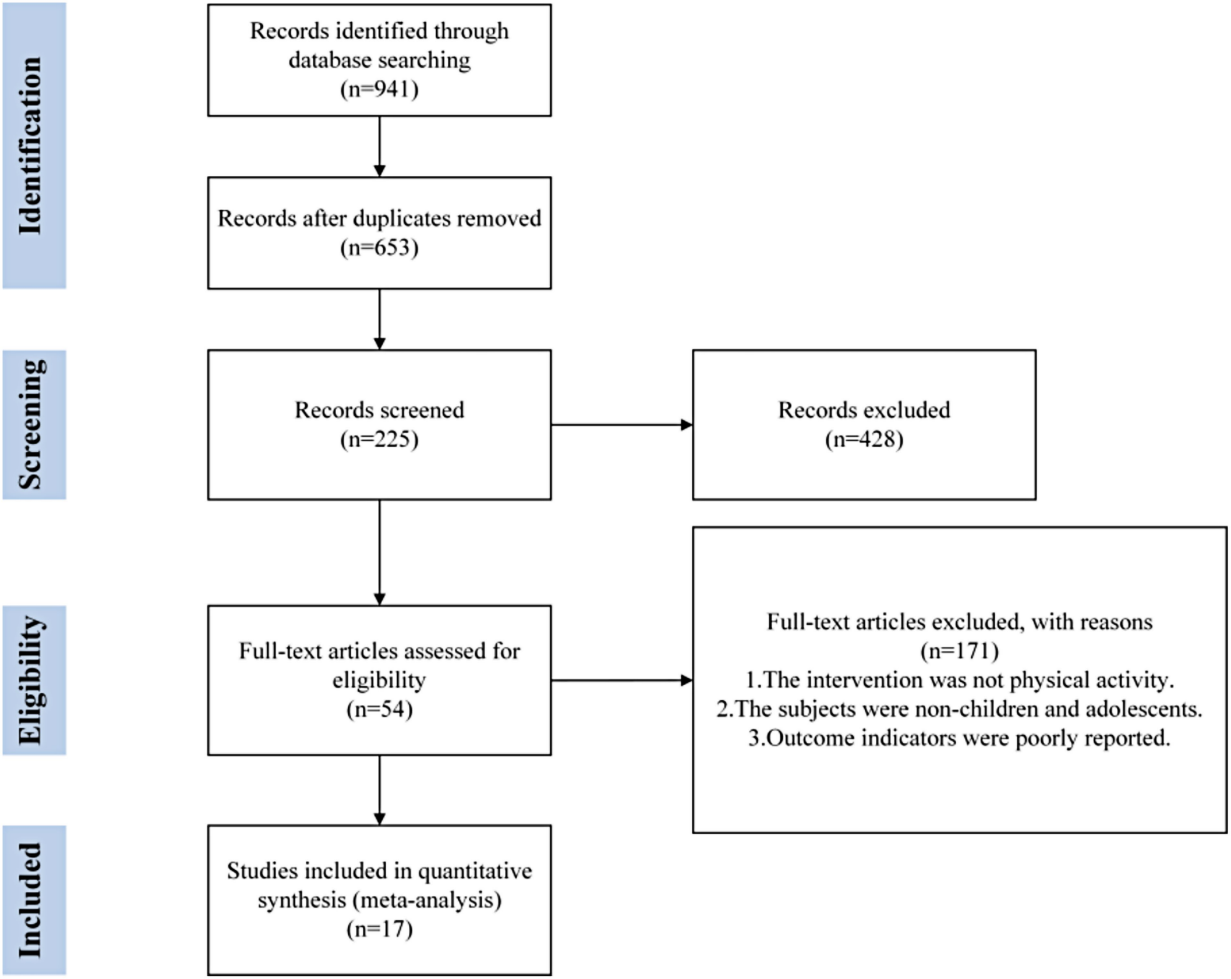
PRISMA flow diagram of the search process for studies.

A total of 17 randomized controlled trials (RCTs) involving 1,357 participants were included. The study selection process is illustrated in Figure 1.

Data extraction included key information such as authors, year of publication, country of study, basic characteristics of participants (e.g., gender, age), intervention modality (e.g., sample size, type of exercise, duration, intensity, frequency), and measurement tools used. Details are given in Table 1. This study delineated these four exercise types based on existing literature and common classifications in the field of sport science: AT, RT, FT, and AT + RT. This division is based on the differences in the effects of different exercises on physical performance and their effectiveness in psychological interventions, and is consistent with the research base of exercise physiology and sports medicine. AT, such as running and swimming, improves whole-body oxygen supply mainly by increasing cardiopulmonary endurance (McCann and Holmes, 1984); RT, such as weightlifting, focuses on building muscle strength (Westcott, 2012); FT, such as yoga and stretching, improves physical and mental health by increasing joint range of motion and muscle elasticity, with mind–body practices like yoga additionally fostering psychological benefits through mindfulness and stress reduction, components that may extend beyond mere flexibility enhancement (Corbin and Noble, 1980); and AT + RT combines the strengths of both, enhancing cardiopulmonary fitness and improving muscle strength (Wang et al., 2025). Therefore, we selected these four exercise types to compare their effects on depressive symptoms in adolescents and examine their dose–response relationships.

**Table 1:** General characteristics of included meta-analyses.

Authors	Nation	Age	Sample		Intervention		type	intensity	Weeks	Min	Per-week	Measurement
			T	C	T	C						
Daley et al. (2006)	UK	11.0 ~ 15.2	27	26	AT	N	Stepping, cycling, rowing	Moderate	8	30	3	CDI
Goldfield et al. (2015)	Canada	15.6 ± 1.4	75	76	AT + RT	N	Treadmills, weight machines	Moderate	22	45	4	BRUMS
Hughes et al. (2013)	USA	17	16	14	AT	P	Treadmills	Moderate	12	30 ~ 40	3	CDRS-R
Jeong et al. (2005)	Korea	16	20	20	AT	P	Dance	Moderate	12	45	3	(SCL)-90
Khalsa et al. (2012)	USA	16.8 ± 0.6	70	30	FT	N	Yoga	Moderate	11	40	3	POMS-SF
Melnyk et al. (2009)	USA	15.5 ± 0.5	11	6	AT	P	Kickball, walking	Moderate	9	50	3	BYI-II
Mohammadi (2011)	Iran	15 ~ 18	40/40	20	AT	N	Soccer, volleyball, table tennis, badminton	Moderate	8	75	3	BDI
Noggle et al. (2012)	USA	17	36	15	FT	P	Yoga	Moderate	10	40	3	POMS-SF
Noorbakhsh and Alijani (2013)	Thailand	19	50	25	AT	N	Soccer, volleyball	Moderate	6	60	3	BDI
Norris et al. (1992)	UK	16.8	14/15/15	16	AT + RT/AT/RT	N	Running, weight training	Moderate and high intensity	10	25 ~ 30	3	MAACL
Petty et al. (2009)	USA	9.35 ± 1.1	27/28	30	AT	N	Running games, jump rope, basketball, and soccer	Moderate and high intensity	13	20	5	RCDS
Philippot et al. (2022)	Australia	17.7	20	20	AT + RT	P	Aerobic group games	Moderate	6	60	4	HADS
Roshan et al. (2011)	Iran	16.9 ± 0.9	12	12	RT	N	Walking on water	Moderate	6	40	3	HAM-D
Wang and Liu (2018)	China	14.0 ± 0.5	90	98	AT + RT	AT	Aerobics and resistance training	Moderate	12	45	4	PHQ-9
Weintraub et al. (2008)	USA	9.9 ± 0.7	9	12	AT	P	Soccer	Moderate and high intensity	24	75	4	CDI
Williams et al. (2019)	USA	9.7 ± 0.9	90	85	AT	P	Running games, ball games, and jump rope	high intensity	32	40	5	CDI
Wunram et al. (2018)	Germany	15.9 ± 1.2	18/17	17	RT/AT	P	Full-body vibration	Moderate	6	30	4	DIKJ

T, experimental group; C, control group. Intervention modality: AT, aerobic exercise; RT, resistance exercise; FT, flexibility training; AT + RT, aerobic combined resistance exercise; P, daily care; N, no intervention. Measurement tools: CDI, Children’s Depression Inventory; BRUMS, Brunel Mood Scale; CDRS-R, Childhood Depression Rating Scale e Revised; BDI, Beck Depression Inventory; MAACL, Multiple Affect Adjective Check List; RCDS, Reynolds Child Depression Scale; HAM-D, Hamilton Depression Rating Scale; DIKJ, Depression List for Children and Adolescents; PHQ-9, Patient Health Questionnaire; POMS-SF, Profile of Mood States short form; HADS, Hospital Anxiety Depression Scale; BYI-II, Beck Youth Inventory (Second Edition); (SCL)-90, Symptom Checklist.

### 3.2 Results of risk of bias evaluation of included studies

**Figure 2:**
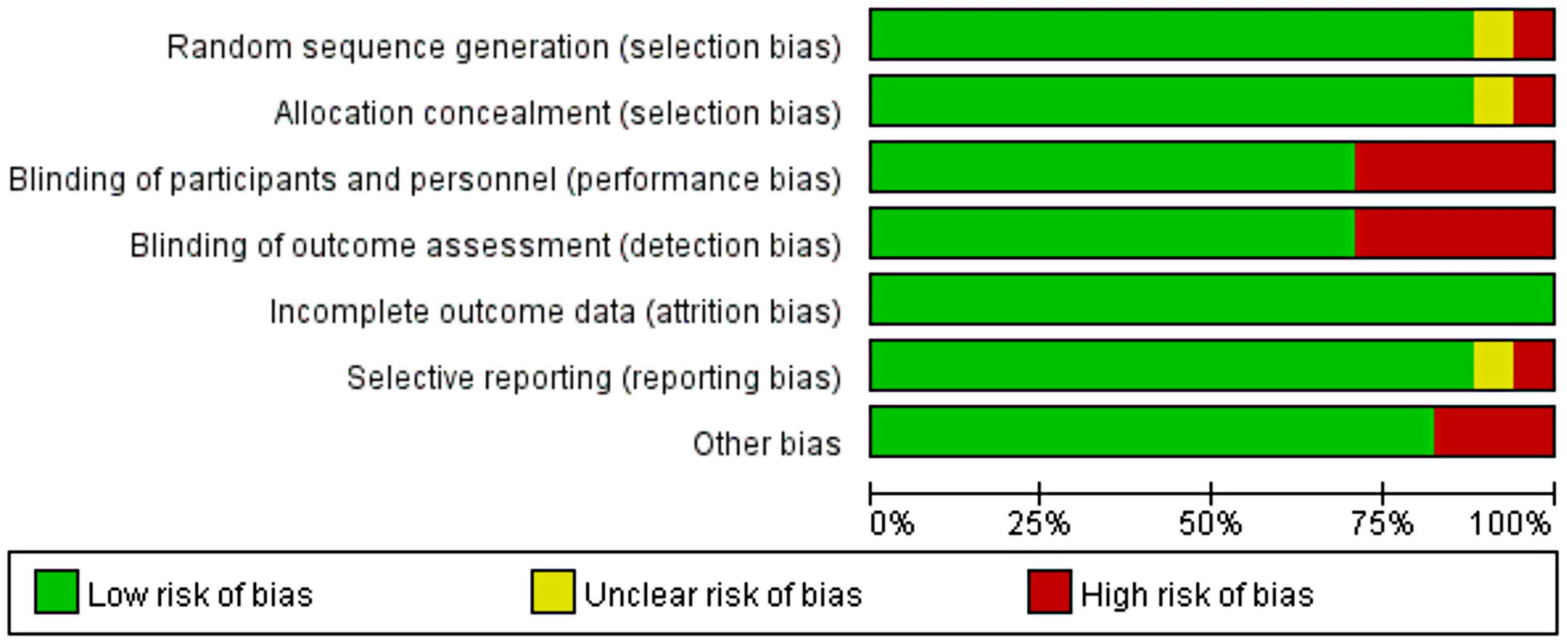
Evaluation of the quality of included studies.

A total of 17 randomized controlled trials (RCT) were included in this study and were critically assessed for risk of bias. The results showed that 2 of the studies (11.8% of the total) were at potential risk of selection bias; 5 studies (29.4% of the total) were at high risk of implementation process and data measurement; and 2 studies (11.8% of the total) were at potential risk of reporting study results. In addition, 3 studies (17.6%) had other forms of bias. Despite the potential for bias in some of the studies, the overall risk of bias was deemed low based on the Cochrane Risk of Bias Tool, which systematically evaluates dimensions like randomization, allocation concealment, and blinding. This indicates that the included literature is of relatively high quality and sufficiently reliable to support subsequent in-depth analyses. The specific results of the risk of bias assessment are detailed in Figure 2. This assessment process helped us to identify potential problems in study design and implementation, but these biases were not found to significantly affect the robustness of the overall conclusions. Therefore, although caution is warranted regarding some of the findings, the overall conclusions of this study are still highly credible and applicable.

### 3.3 Overall analysis of the effectiveness of exercise as an intervention for depression in children and adolescents

**Figure 3:**
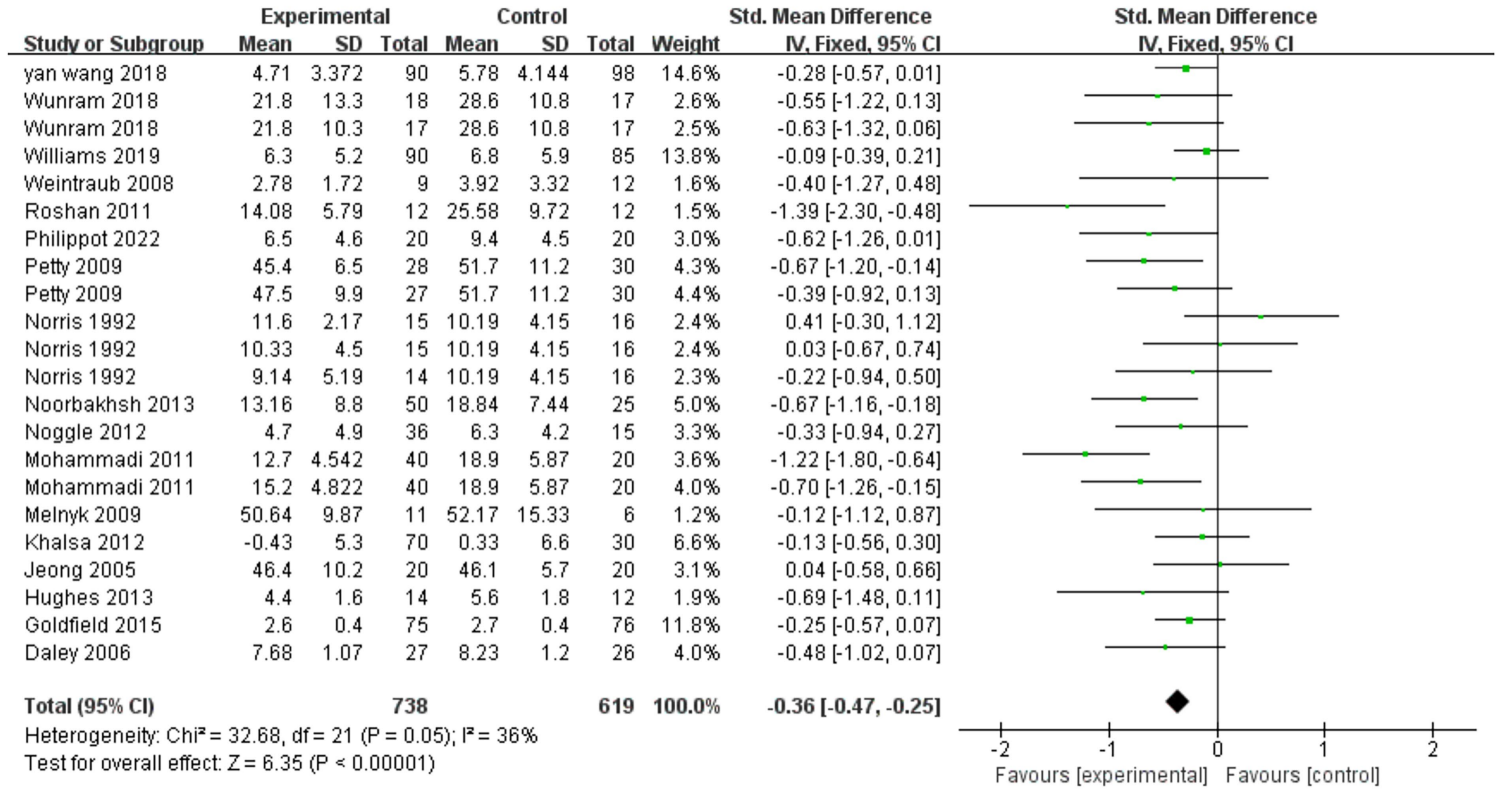
Forest plot of the effect of exercise on depression intervention in children and adolescents.

A test of heterogeneity of outcome indicators for exercise intervention for depression in children and adolescents found (Figure 3) that overall exercise had a significant effect on depression in children and adolescents (SMD = −0.36, 95% CI: −0.47 to −0.25, *P* < 0.001) suggesting low heterogeneity (*I^2^* < 50%, *p* > 0.05), which strengthens the validity and reliability of the overall findings, This suggests that despite the potential risks associated with some of the studies, as well as differences in study methodology, interventions and sample characteristics, these differences did not significantly affect the overall intervention effect. The results of the heterogeneity test further confirmed that the variability between the included studies did not diminish the positive effect of exercise on alleviating depressive symptoms. This suggests that exercise intervention as an intervention can have a generalized positive effect on depressive symptoms in children and adolescents across different study contexts and sample characteristics. Although the overall analysis confirmed the effectiveness of exercise, the optimal modality, dose, and frequency still require further investigation.

### 3.4 Network meta-analysis results

#### 3.4.1 Network relations and coherence analysis

**Figure 4:**
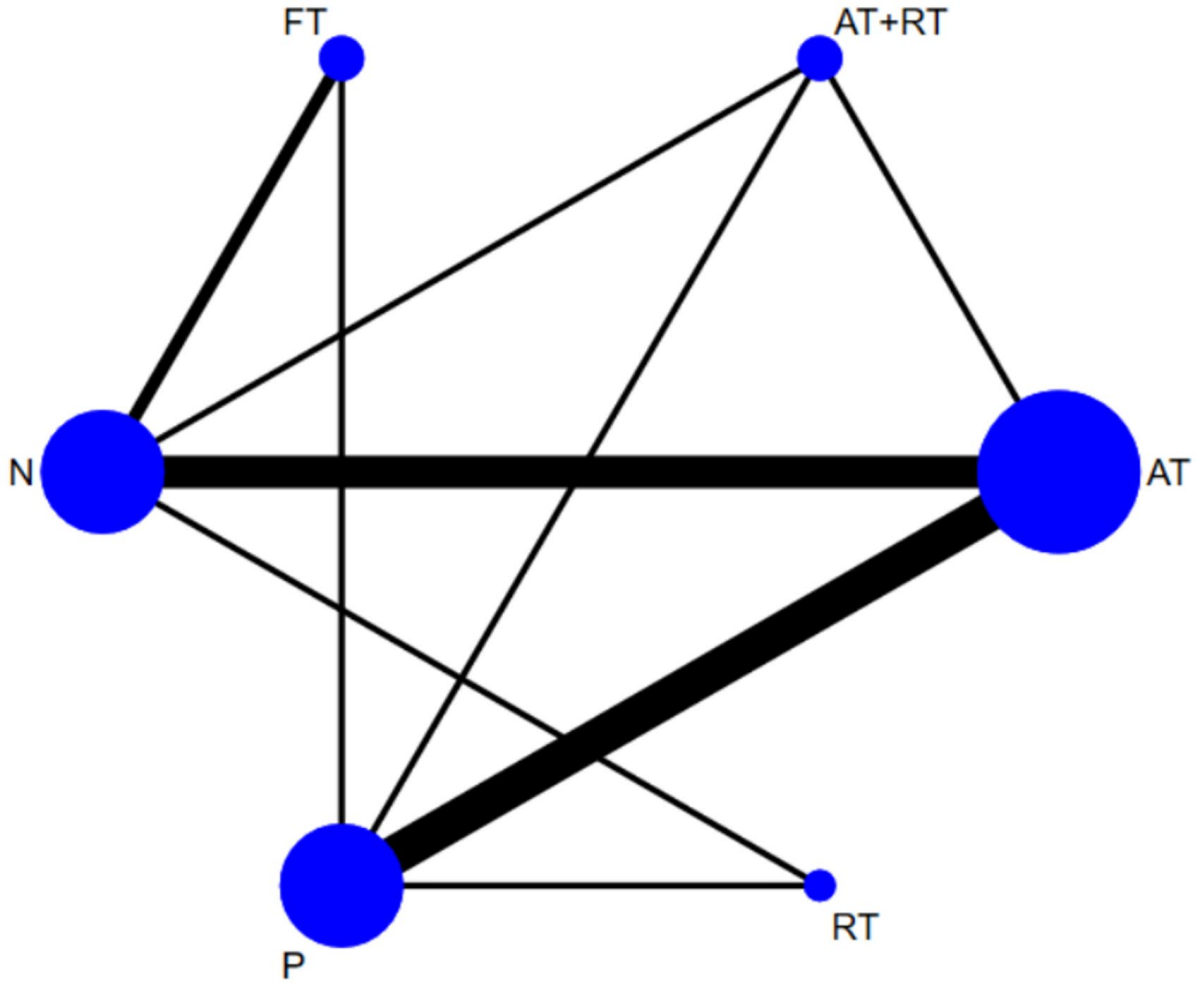
Network correlations for the effect of exercise on depression intervention in adolescents.

In an in-depth analysis of the effects of different exercise interventions, this study found that 13 different two-by-two comparisons could be formed between the six interventions. These six include the four explicitly defined earlier—AT, RT, FT, and AT + RT, as well as two additional conditions: where “P” refers to a placebo intervention and “N” denotes no intervention. In order to show these comparison relationships more clearly, the study constructed a network relationship graph based on Bayesian modeling as shown in Figure 4. In the graph, the nodes represent exercise interventions, and lines indicate direct comparisons between them. Based on the analysis, a total of nine direct comparisons of evidence were found, and these direct comparisons provided a clear comparison of the differences in effectiveness between the various exercise interventions.

In this study, a consistency model was employed for the network meta-analysis to estimate the relative effects of different exercise interventions. In addition to direct comparisons, indirect comparisons were formed through one or more intermediate interventions, allowing for inference of relative effects even in the absence of head-to-head trials. A loop-specific inconsistency test was conducted to assess the coherence between direct and indirect evidence. The inconsistency factor (IF) values for each closed loop ranged from 0.01 to 0.84. Following the commonly accepted threshold in network meta-analysis (IF < 1.0 indicates acceptable inconsistency), these values suggest good consistency across the network, with 0.84 remaining within the range of acceptable consistency. This network structure not only facilitates an intuitive understanding of the comparative effectiveness of multiple interventions but also enhances the robustness of our findings by integrating all available evidence.

By integrating direct and indirect comparative evidence, this study provides important clues and rationale for comprehensively evaluating the effectiveness of various exercise interventions, and provides a reliable basis for subsequent optimization and selection of more effective exercise intervention strategies.

#### 3.4.2 Pairwise meta-analyses

In comparing the efficacy of various exercise interventions for alleviating depressive symptoms in children and adolescents, RT, AT, and AT + RT each demonstrated significant superiority over the control group (detailed in Table 2). Specifically, RT (SMD = −0.52, 95% CI: −0.95 to −0.09) exhibited the largest effect size, followed by AT (SMD = −0.40, 95% CI: −0.56 to −0.25) and AT + RT (SMD = -0.30, 95% CI: −0.49 to −0.10), with all interventions achieving statistical significance (*P* < 0.05). Conversely, FT (SMD = −0.20, 95% CI: −0.55 to 0.15; *P* > 0.05) did not yield a statistically significant improvement in depressive symptoms among participants.

**Table 2:** Details of pairwise meta-analyses.

Comparison	Number of studies	SMD	95%CI	*I* ^2^	*P*
AT vs. Control	11	−0.40	(−0.56, −0.25)	49%	<0.001
RT vs. Control	3	−0.52	(−0.95, −0.09)	66%	<0.05
AT + RT vs. Control	4	−0.30	(−0.49, −0.10)	0%	<0.01
FT vs. Control	2	−0.20	(−0.55, 0.15)	0%	0.26

These findings suggest that both RT, AT, and AT + RT can serve as effective adjuvant therapies for improving depressive symptoms in children and adolescents, with RT demonstrating the highest efficacy among the modalities evaluated. This supports the potential generalization of RT in clinical settings for adolescent depression management. While FT did not yield statistically significant improvements in this analysis, their efficacy in specific subgroups or under alternative intervention parameters cannot be excluded. Future research should explore contextual factors influencing treatment response, such as age stratification, baseline symptom severity, and adherence rates. In conclusion, RT, AT, and AT + RT emerge as evidence-based interventions for alleviating depressive symptoms in youth, with RT showing particular promise as a primary or adjunctive treatment modality.

#### 3.4.3 Best probability ranking results

The SUCRA values based on the probability ranking show that RT (99.7%) > AT (67.5%) > AT + RT (59.8%) > FT (35.5%) > P (26.7%) > N (10.7%), and that RT had the highest SUCRA value (99.7%), suggesting it may be the most effective intervention among those studied. This result emphasizes the importance of RT in mental health interventions that may provide children and adolescents with an effective way of emotional support and stress reduction. This was followed by AT, which had a SUCRA value of 67.5%. Combined with the network relationship analysis, it can be seen that AT, being widely used, may offer a more accessible option for various age groups, and may help adolescents reduce symptoms of anxiety and depression, according to previous evidence. AT + RT was ranked third with a SUCRA value of 59.8%, which, although its effectiveness is slightly lower than that of RT and AT alone, still shows good potential for a combined intervention, and this combined intervention may provide a more flexible exercise program for individuals with different needs. In addition, FT had a SUCRA value of 35.5%, which was a more limited improvement but still had some positive effects in terms of overall health and physical and mental coordination. P had a SUCRA value of 26.7%, which showed a less than expected effect in terms of improvement in depressive symptoms, and N with a SUCRA value of only 10.7%, indicating it was least likely to be effective.

**Figure 5:**
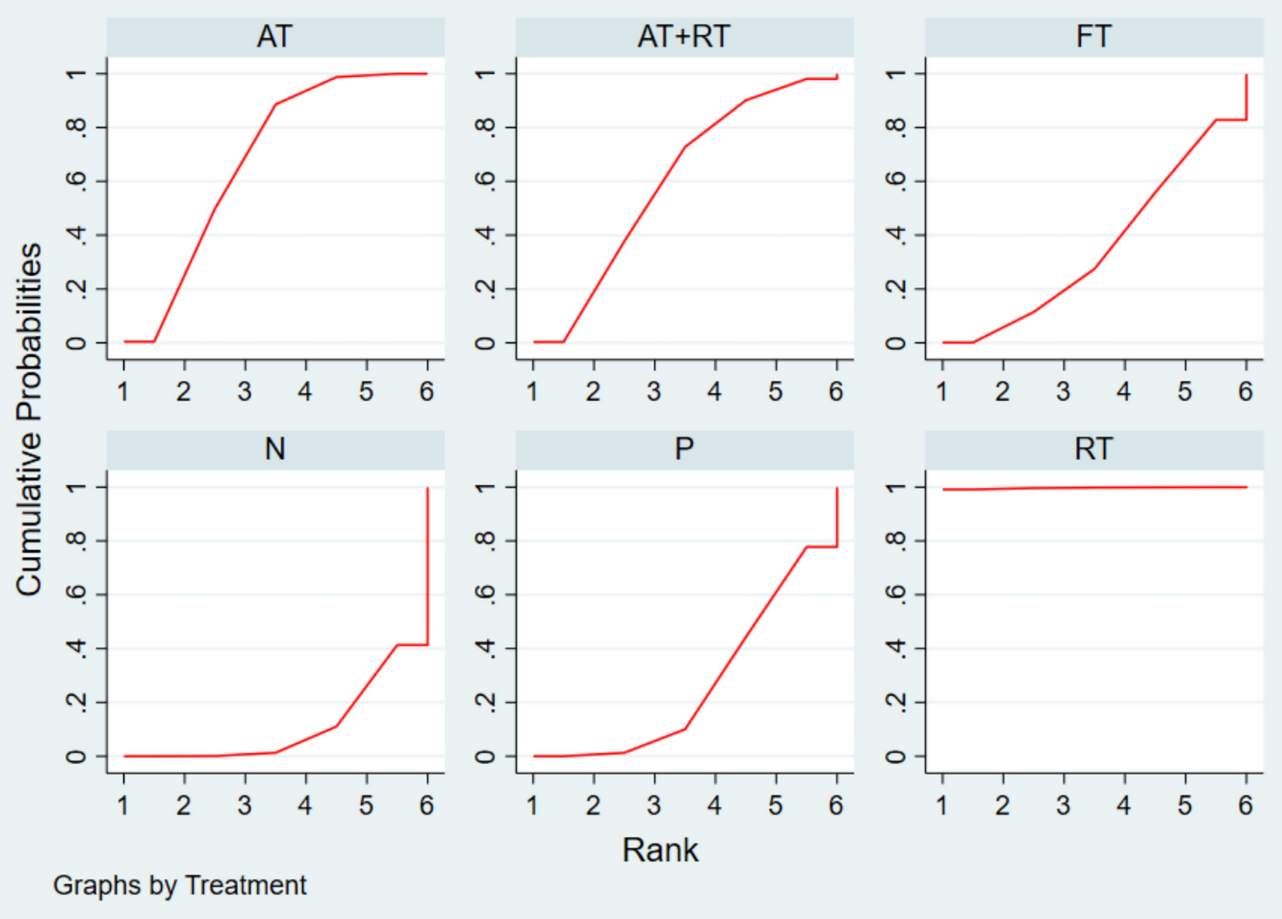
Probability ranking plot of the effect of exercise on depression intervention in children and adolescents.

Taken together, RT and AT are likely preferred for alleviating depressive symptoms in children and adolescents, emphasizing the positive role of exercise interventions in promoting mental health. These findings may offer useful guidance for designing exercise-based interventions in clinical and community settings. Detailed data can be found in Figure 5 and Table 3.

**Table 3:** Ranking of the effectiveness of exercise on the effect of intervention on depression in children and adolescents.

Intervention	Sucra (%)	Best	Rank
RT	99.7	99.1	1.0
AT	67.5	0.4	2.6
AT + RT	59.8	0.3	3.0
FT	35.5	0.1	4.2
P	26.7	0	4.7
N	10.7	0	5.5

“Best” refers to the top-ranked position in the analysis, and “Rank” denotes the probability (%) associated with this position.

### 3.5 Dose analysis of exercise to improve depressive symptoms in children and adolescents

**Figure 6:**
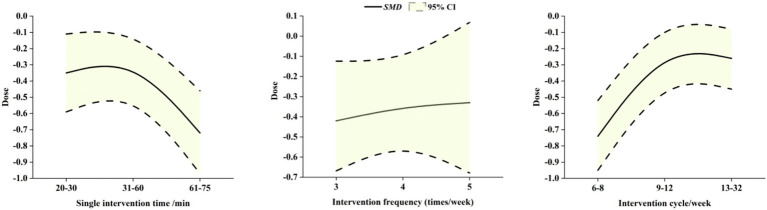
Dose–response relationship of exercise intervention effect on depression in children and adolescents.

To investigate the dose of exercise on improving depressive symptoms in children and adolescents, the effect of exercise interventions was profiled by grouping them according to single exercise duration, exercise frequency, and intervention period, which in turn revealed the complex dose relationship between exercise and depressive symptoms in children and adolescents (see Figure 6).

To clarify dose–response relationships, we explicitly defined optimal dose and minimum dose of exercise interventions for reducing depressive symptoms in young people, based on aggregated data from included studies. Minimum dose was defined as the smallest duration/frequency at which the pooled effect size (SMD) became statistically significant (*P* < 0.05). Optimal dose was identified as the threshold where effect sizes plateaued. First, on the dimension of single exercise duration, it was found that a single exercise session of 20 to 30 min (SMD = −0.35, 95% CI: −0.59 to −0.11, *P* < 0.01) appears to represent the lowest dose of exercise intervention to improve depressive symptoms in child adolescents, whereas a single exercise session controlled between 61 and 75 min (SMD = −0.72, 95% CI: −0.97 to −0.42, *P* < 0.001) was associated with the most significant reduction in depressive symptoms in child adolescents, a finding that emphasizes the positive effect of controlling the duration of moderate exercise on improving depression. Secondly, in terms of exercise frequency, the results showed that 3 sessions of exercise per week (SMD = −0.42, 95% CI: −0.67 to −0.16, *P* < 0.01) tends to be the most effective dose to improve depressive symptoms in children and adolescents, and that when the frequency of exercise per week was increased to 5 sessions (SMD = −0.33, 95% CI: −0.68 to 0.02, *P* > 0.05), the intervention effect did not increase and was not significant, which may have introduced additional psychological stress, potentially reducing the intervention’s positive effects. Finally, according to the analysis of the intervention period, 6–8 weeks (SMD = −0.74, 95% CI: −0.95 to −0.52, *P* < 0.001) emerges as the optimal dose of exercise to improve the depressive symptoms of the children and adolescents. Meanwhile, significant improvement remained as the intervention period was extended to 13–32 weeks (SMD = −0.26, 95% CI: −0.45 to −0.08, *P* < 0.01).

This finding provides empirical support for the development of a long-term exercise intervention program and emphasizes the importance of sustained intervention for the mental health of children and adolescents. The above dosage analysis reveals the intervention effect of exercise intervention on improving depressive symptoms in children and adolescents, and offers useful insights for tailoring exercise intervention programs to manage depressive symptoms in children and adolescents.

### 3.6 Sensitivity analysis, consistency assessment, and publication bias evaluation

Sensitivity analyses were performed by sequentially excluding each included study and re-computing the pooled effect size. The results showed no significant alteration in the overall effect estimate after individual study exclusion, indicating that the meta-analysis findings were robust.

For inconsistency assessment, both global and local tests were conducted. The results revealed no statistically significant inconsistency in outcome indicators (*p* > 0.05), suggesting low heterogeneity in inconsistency across studies. Evidence from direct and indirect comparisons was generally consistent, supporting the comparability of the estimated effects of different interventions.

**Figure 7:**
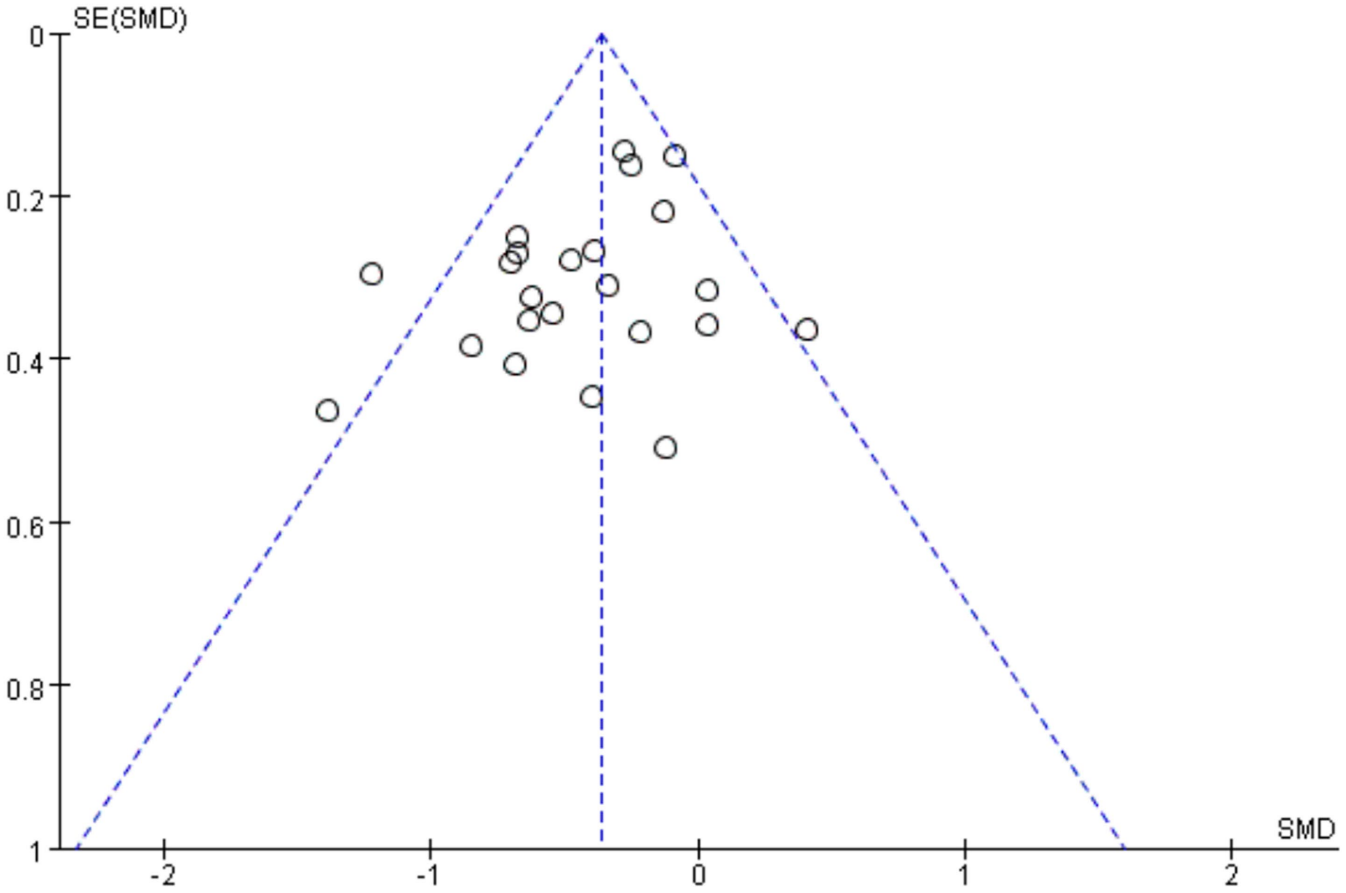
Funnel plot of the effect of exercise intervention on depression in children and adolescents.

To assess the potential for publication bias in the analysis of multiple exercise interventions on depressive symptoms in children and adolescents, a funnel plot was constructed (Figure 7). The funnel plot, constructed based on standard errors, showed that most studies were distributed symmetrically around the central line, suggesting a low risk of small-study effects influencing the results. This indicates that the results were not systematically biased due to sample size limitations, even though small-study effects can exist independently of significant bias in tests. In addition to the visual assessment, we conducted statistical tests to evaluate publication bias more objectively. Egger’s regression test was performed and showed no significant evidence of publication bias (*p* = 0.32). Similarly, Begg’s test yielded non-significant results (*p* = 0.28), further supporting the symmetry observed in the funnel plot.

These findings suggest that the included studies were not systematically excluded or selectively published based on the significance of their results. Therefore, the credibility and stability of the meta-analytic outcomes are considered high, and the overall quality of the evidence base is relatively balanced. The absence of significant publication bias provides a solid foundation for the interpretation of the effectiveness of exercise interventions in reducing depressive symptoms among children and adolescents.

## 4 Discussion

This study employed a comprehensive dose–response network meta-analysis to examine the relationship between physical activity and depressive symptoms in adolescents. Building on these foundational data, our research synthesized evidence across diverse exercise modalities, including aerobic (AT), resistance (RT), flexibility (FT), and combined aerobic-resistance (AT + RT) training to assess their comparative efficacy. The analysis supports the emerging consensus that RT may confer particularly pronounced benefits for mood regulation in youth. This could be attributed to both physiological and psychological mechanisms: physiologically, RT has been shown to stimulate the release of endorphins and regulate hypothalamic–pituitary–adrenal (HPA) axis activity, which are key in alleviating negative emotions (Hamedinia et al., 2017); psychologically, the sense of accomplishment from progressive overload in RT and improved self-efficacy may further enhance mood states (Dow et al., 2025).

Exercise may be an effective approach to alleviating depressive symptoms in adolescents, and the current findings reinforce previous evidence supporting the effectiveness of exercise interventions for adolescent depression. Previous research has highlighted the significant impact of exercise on depression, with potential mechanisms including increased release of β-endorphins (Kleppang et al., 2018), enhanced availability of brain neurotransmitters (e.g., serotonin, dopamine, and noradrenaline) (Radovic et al., 2018), and elevated levels of brain-derived neurotrophic factors. Additionally, exercise has been linked to improvements in self-esteem, self-perception, and a sense of personal accomplishment. Moreover, exercise offers opportunities for social interaction, which is especially important for adolescents who often experience feelings of loneliness and isolation. Participation in exercise groups or fitness classes can help adolescents build social support networks and foster meaningful connections, which are crucial for maintaining mental well-being (Vella, 2019). Without considering exercise dosage, our findings suggest that RT may be the most effective modality for alleviating depressive symptoms in adolescents. The unique advantages of RT in improving adolescent mental health have also been previously documented in the literature.

However, some previous meta-analyses focusing on older populations have ranked the efficacy of exercise interventions for depression as follows: AT + RT > RT > AT (Miller et al., 2020). This discrepancy may be attributed to age-related confounding factors such as cognitive decline, which can influence individuals’ capacity to adhere to more complex or physically demanding exercise protocols like RT. In older adults, the combined AT + RT interventions may strike a balance between cardiovascular and muscular benefits, thus showing superior effects. In contrast, the present study focused exclusively on children and adolescents, a group with distinct physiological and psychological characteristics. For younger individuals, which may hypothetically reduce adherence, although this requires further investigation. Furthermore, especially in populations not accustomed to structured training, the potential for exercise-induced fatigue in combined programs may blunt the psychological benefits, especially if recovery is insufficient. These factors could partially explain why AT + RT did not demonstrate superior efficacy compared to RT or AT alone in this adolescent population. Nonetheless, the interpretation of these findings should be approached with caution. Future studies—specifically age-stratified RCTs that directly compare combined and single-modality interventions, may be needed to further explore the differential effects of combined versus single-mode exercise interventions across various age groups.

Regarding exercise duration, the analysis found that 20–30 min of exercise per session was the lowest effective dose to improve depressive symptoms in adolescents. An optimal exercise duration of 61–75 min was associated with the most significant improvement in depressive symptoms. This finding was derived from subgroup analyses, which specifically examined the effects of different exercise durations; among these, the 61–75 min subgroup included data from studies, providing empirical support for this association. This is partially consistent with findings from Wang et al. (2022), whose research specifically demonstrated that 30-min aerobic exercise sessions yielded the best intervention effects for adolescents with depression, supporting the efficacy of 30-min bouts within our identified minimum effective range. In terms of exercise frequency, three sessions per week was found to be most effective for alleviating depressive symptoms, consistent with data from Wang et al. (2022) showing that thrice-weekly interventions outperformed both lower and higher frequencies. Notably, our finding that increasing frequency to five times per week did not enhance benefits mirrors Zhou et al.’s (2024) observation that excessive exercise frequency may induce psychological stress, thereby attenuating intervention effects. For intervention duration, 6–8 weeks produced the most pronounced reductions in depressive symptoms, while extended interventions remained effective. This is supported by Yan et al. (2024), whose longitudinal analysis demonstrated sustained benefits of long-term exercise, reinforcing the value of maintaining exercise adherence beyond initial intervention phases.

These findings provide an important reference for healthcare practitioners and adolescents, highlighting the importance of tailoring exercise interventions to individual preferences, capacities, and mental health status. Incorporating a variety of effective exercise modalities and clearly defined dosages allows clinicians to tailor recommendations to each individual’s preferences, physical abilities, and specific mental health needs. This individualized exercise prescription may ensures the most effective reduction of depressive symptoms in adolescents. However, it is critical to distinguish between remission of depressive symptoms and a formal diagnosis of depression. While the Minimum Clinically Important Difference (MCID) provides a useful metric for assessing meaningful clinical improvement, defined as the smallest change in symptoms that patients or clinicians perceive as beneficial (Jaeschke et al., 1989), a statistically significant reduction in symptoms does not necessarily equate to a complete remission of depression. Complete remission, as specified by the Diagnostic and Statistical Manual of Mental Disorders, Fifth Edition (DSM-5), requires the absence of all depressive symptoms for at least 2 weeks, with no significant functional impairment (American Psychiatric Association, 2013). This distinction is essential for both clinical decision-making and the development of public health recommendations. Misinterpreting symptom remission as recovery may lead to type I errors, which may result in incorrect inferences about treatment efficacy. Future research should aim to elucidate the long-term effects of exercise on depression, particularly by distinguishing between short-term symptom remission and long-term changes in diagnostic status. Such research is essential to fully understand the role of exercise in the management of adolescent depression and to develop treatment programs that may be effective and tailored to the individual needs of patients.

In this study, a network Meta-analysis was used to compare and assess the exercise modality and dosage with the best intervention effect to improve the outcome of depression in children and adolescents. Network Meta-analysis was performed using the network package in Stata 16.0 software, which uses frequency statistics that have been proven to be accurate and reliable in numerous studies and are widely used in medicine and nursing. However, there are still some limitations mainly reflected in the following two aspects: (1) Although this study adopted the methods of literature quality assessment, heterogeneity test, consistency test, etc., and sought to maximize the avoidance of interference from potential factors such as sample characteristics, experimental design, and dosage control, it is still difficult to completely eliminate all the biasing factors due to the limitations of the available data and methodology, which may have affected the precision of some results. (2) This study included four representative exercise types (AT, RT, FT, AT + RT) based on prior research, but unregulated intensity variations within these modalities may mask nuanced effects, for example, moderate- vs. vigorous-intensity AT likely differ in psychological impact (Yan et al., 2024), and varying RT loads could affect depression-linked stress hormones, introducing unmeasured heterogeneity (Li et al., 2024). Excluding other types limits breadth, as these may offer unique benefits via mindfulness or social connectedness (Kok and Singer, 2017), while lacking standardized intensity metrics across interventions hinders efficacy comparisons (Yan et al., 2024).; Therefore, future research should explore additional modalities such as dance, team sports, or mind–body practices.

## 5 Conclusion

By systematically evaluating the effects of various exercise modalities on improving depressive symptoms in children and adolescents, this study found that both RT, AT, and AT + RT significantly alleviated depressive symptoms, within the limits of the available data and methodological variability. These findings suggest that exercise interventions may have meaningful positive impacts on the mental health of young individuals. Based on the results, it is recommended that children and adolescents engage in exercise interventions lasting 20–30 min, three times per week, for a minimum duration of 6–8 weeks. This regular frequency and duration of physical activity appear to be beneficial for reducing depressive symptoms and may contribute to the prevention and management of adolescent depression.

## Data Availability

The original contributions presented in the study are included in the article/supplementary material, further inquiries can be directed to the corresponding author.
