# Supplementary material for: Comparative effects of exercise type and dose on depression in children and adolescents: a network meta-analysis
Source: Front Psychol. 2025 Aug 13;16:1632111. doi: 10.3389/fpsyg.2025.1632111 (PMC12381835; doi:10.3389/fpsyg.2025.1632111)
Supplement: Supplementary file 1 [file Data_Sheet_1.docx]

**Comparative Effects of Exercise Type and Dose on Depression in Children and Adolescents: A Network Meta-Analysis**

[Supplementary file: Search strategy](#_heading=h.17dp8vu)

Pubmed

((exercis*[Title/Abstract] OR aerobic*[Title/Abstract] OR running[Title/Abstract] OR jogging[Title/Abstract] OR walk*[Title/Abstract] OR hiking[Title/Abstract] OR swim*[Title/Abstract] OR aquatic*[Title/Abstract] OR cycling[Title/Abstract] OR bicycl*[Title/Abstract] OR strength*[Title/Abstract] OR activit*[Title/Abstract] OR fitness[Title/Abstract] OR physical medicine[Title/Abstract] OR resistance[Title/Abstract] OR lift*[Title/Abstract]) OR yoga[Title/Abstract] AND (depression[Title/Abstract] OR dysthymia[Title/Abstract])) AND (randomized controlled trial[Title/Abstract] OR clinical trial[Title/Abstract])

PsychINFO

S1 SU depression OR SU dysthymia OR SU depressive disorder

S2 SU randomized controlled trials OR SU clinical trial OR SU rct

S3 SU exercise OR SU aerobic OR SU running OR SU jogging OR SU walk OR SU hiking OR SU swim OR SU aquaSUc OR SU strength OR SU resistance OR SU yoga

S4 (SU exercise OR SU aerobic OR SU running OR SU jogging OR SU walk OR SU hiking OR SU swim OR SU aquaSUc OR SU strength OR SU resistance OR SU yoga) AND (S1 AND S2 AND S3)

scopus

#1 (TITLE-ABS-KEY(exercise) OR TITLE-ABS-KEY(aerobic) OR TITLE-ABS-KEY(running) OR TITLE-ABS-KEY(Jogging) OR TITLE-ABS-KEY(Walk) OR TITLE-ABS-KEY(hiking) OR TITLE-ABS-KEY(Swim) OR TITLE-ABS-KEY(Aquatic) OR TITLE-ABS-KEY(Cycling) OR TITLE-ABS-KEY(Bicycl) OR TITLE-ABS-KEY(strength) OR TITLE-ABS-KEY(Activit) OR TITLE-ABS-KEY(fitness) OR TITLE-ABS-KEY(train) OR TITLE-ABS-KEY(physical medicine) OR TITLE-ABS-KEY(Resistance) OR TITLE-ABS-KEY(lift) OR TITLE-ABS-KEY(yoga))

#2(TITLE-ABS-KEY(depression) OR TITLE-ABS-KEY(dysthymia))

#3(TITLE-ABS-KEY(randomized controlled trial) OR TITLE-ABS-KEY(clinical trial))

#4 #1 AND #2 AND #3

Embase

#1

'exercise'/exp OR exercise OR aerobic:ab,ti OR running:ab,ti OR jogging:ab,ti OR walk:ti OR hiking:ab,ti OR swim:ab,ti OR aquatic:ab,ti OR cycling:ab,ti OR bicycl:ab,ti OR strength:ab,ti OR activit:ab,ti OR fitness:ab,ti OR 'physical medicine':ab,ti OR resistance:ab,ti OR lift:ab,ti OR yoga:ab,ti

#2

'depression'/exp OR depression OR dysthymia:ab,ti

#3

'randomized controlled trial'/exp OR 'randomized controlled trial' OR 'clinical trial':ab,ti

#4

#1 AND #2 AND #3

SportDiscus

S1 TI depression OR TI dysthymia OR TI depressive disorder

S2 TI randomized controlled trials OR TI clinical trial OR TI RCT

S3 TI exercise OR TI aerobic OR TI running OR TI jogging OR TI walk OR TI hiking OR TI swim OR TI aquaTIc OR TI strength OR TI resistance

S4 S1 AND S2 AND S3

Web of science

(TS=(("physical activity" OR exercise OR "aerobic training" OR "resistance training" OR "flexibility training" OR "yoga" OR "tai chi") AND (depress* OR "depressive symptoms" OR "depressive disorder")) AND TS=(child* OR adolescen* OR teen* OR youth OR "young people") AND TS=("randomized controlled trial" OR RCT OR "intervention study"))

MEDLINE

("physical activity"[MeSH Terms] OR "physical activity"[All Fields] OR exercise[All Fields] OR "aerobic training"[All Fields] OR "resistance training"[All Fields] OR "flexibility training"[All Fields] OR yoga[All Fields] OR "tai chi"[All Fields]) AND (depress*[All Fields] OR "depressive symptoms"[All Fields] OR "depressive disorder"[MeSH Terms] OR "depressive disorder"[All Fields]) AND (child*[All Fields] OR adolescen*[All Fields] OR teen*[All Fields] OR youth[All Fields] OR "young people"[All Fields]) AND ("randomized controlled trial"[Publication Type] OR RCT[All Fields] OR "intervention study"[All Fields])

CENTRAL

(("physical activity" OR exercise OR "aerobic training" OR "resistance training" OR "flexibility training" OR yoga OR "tai chi") AND (depress* OR "depressive symptoms" OR "depressive disorder") AND (child* OR adolescen* OR teen* OR youth OR "young people") AND ("randomized controlled trial" OR RCT OR "intervention study"))
